# The reliability conundrum of DWI-ASPECTS in stroke assessment: a systematic review of agreement on DWI-ASPECTS in anterior circulation stroke

**DOI:** 10.3389/fneur.2026.1900599

**Published:** 2026-10-23

**Authors:** Mobin Jamal, Vincent Brissette, Gabriele Zitikyte, Michel Shamy, Robert Fahed

**Affiliations:** 1 Department of Medicine (Neurology), The Ottawa Hospital, Ottawa, ON, Canada; 2 Ottawa Hospital Research Institute, University of Ottawa, Ottawa, ON, Canada

**Keywords:** DWI-ASPECT, DWI-ASPECTS, ischemic stroke, reliability, stroke

## Abstract

**Background:**

The DWI-ASPECT score (DWI-ASPECTS) can aid patients’ selection for thrombectomy. This systematic review aimed to evaluate clinicians’ interrater and intrarater agreement on DWI-ASPECTS for anterior circulation stroke patients.

**Methods:**

We conducted a broad search of electronic databases (CINAHL, EBM Reviews, MEDLINE, EMBASE & EMBASE Classic) between January 1st, 2000, and September 9^th^, 2026. Our primary outcome was to collect data on any available measures of agreement for DWI-ASPECTS. Measures of agreement collected included ICC (intraclass correlation coefficient), weighted or unweighted kappa.

**Results:**

Out of 3,658 articles screened, nineteen studies reporting 37 measures of agreement were included in the review. Populations, methods, analyses, and results were heterogeneous (poor to excellent agreement), precluding a meta-analysis. For interrater agreement, measures of agreement using full scale ICC ranged from strong to excellent, ranged from poor to strong using unweighted kappa, and ranged from moderate to excellent using weighted kappa. Only four studies offered interrater agreement values on DWI-ASPECTS<6. Only two studies offered information on intrarater agreement values.

**Conclusion:**

Data on the reliability of DWI-ASPECTS in acute ischemic stroke is scant, while the methodologies and results in the studies assessed are heterogeneous. More robust studies are required, particularly for strokes with DWI-ASPECTS <6.

## Introduction

The Alberta Stroke Program Early CT Score (ASPECTS or ASPECT score), first described in 2000, (1) is a 10-point categorical scale designed to quantify early signs of ischemia on computed-tomography (CT) pictures of patients with acute ischemic stroke. The purpose was to help in acute stroke management and predict prognosis of stroke patients treated with intravenous thrombolysis (1). The ASPECT score has since been used as a vital patient selection criterion for thrombectomy trials (2–5) and is now included in various national stroke guidelines in their hyperacute treatment algorithm to aid in thrombectomy decisions (6–8). Despite its large-scale use and impact on treatment decisions, it has been previously demonstrated that interrater and intrarater agreement for ASPECTS on CT scan has not been extensively studied, and data suggest overall modest agreement (9).

The ASPECT score can also be used on Magnetic Resonance Imaging (MRI) images of acute ischemic stroke patients. More specifically, the Diffusion-Weighted Imaging (DWI) sequence allows visualization of hyperacute ischemia and can be used for ASPECT scoring (DWI-ASPECTS). The DWI-ASPECT score allows for more sensitive detection of early ischemic changes (10), and has been associated with lower mortality at 90 days compared to CT scan (11). It is also widely used in France as the initial diagnostic tool for acute ischemic stroke (11) and is endorsed as an initial diagnostic modality by the American Heart Association, the American Stroke Association (12), and through the European Stroke Organisation/ European Society for Minimally Invasive Neurological Therapy (ESO/ESMITN) guidelines (8). While thrombectomy was traditionally indicated for patients with ASPECTS ≥ 6 (6–8), recent randomized controlled trials have started to demonstrate improved outcomes in patients treated with thrombectomy with a larger infarct burden (ASPECTS 3–5), allowing for treatment of patients with larger stroke size (13–16). The DWI-ASPECTS has the potential to be more widely used in this setting, given its higher sensitivity for identifying the fullest extent of infarct (17). Therefore, given the increasing clinical and research interests regarding the use of DWI-ASPECTS, it is crucial to systematically evaluate agreement for DWI-ASPECTS.

## Methods

We conducted this systematic review according to the Preferred Reporting Items for Systematic Reviews and Meta-Analysis guideline (18).

### Eligibility criteria

This systematic review aimed to include studies meeting the following inclusion criteria: studies assessing the interrater and/or intrarater agreement of DWI-ASPECTS on hyperacute MRI imaging (i.e., within 24 h of onset) of patients diagnosed with an acute ischemic stroke of the anterior circulation. We excluded studies focused solely on the posterior circulation, studies for whom patients were assigned a CT-ASPECT or CTP-ASPECT score solely, and studies in languages other than English or French. Included studies had to offer any measures of intrarater or interrater agreement on DWI-ASPECTS for anterior circulation stroke patients. We excluded review articles, abstracts without full text, protocols, and supplements.

### Information sources and search strategy

We conducted a broad search of various electronic databases (MEDLINE, EMBASE & EMBASE Classic, EBM Reviews and CINAHL) for published articles between January 1st, 2000, and September 9th, 2026. Prior to starting our review process, our team consulted with a librarian who created the search strategy. Please refer to the Supplementary Table 1 for our search strategy.

### Selection process

Overall, 7,649 studies were identified from our search of all databases. After we removed duplicates, 3,658 studies had to be screened. First, through the Covidence Software Program, two independent reviewers screened the titles and abstracts of all studies to identify those meeting inclusion criteria. Discrepancies between the two reviewers were resolved by consensus first, and by a third independent reviewer if consensus was not reached. Second, the same two independent reviewers screened the references of all included articles to identify missed studies from our initial screen. Finally, the two reviewers screened the full text of all eligible articles. Discrepancies were resolved in the same way as for the first step of the screening process.

### Data collection and data items

Data extraction from each included study was performed with the Excel software. We used a data collection form created by the research group and refined it as needed. Our primary outcome was to collect data on any available measures of agreement for DWI-ASPECTS. We also collected descriptive data from each published article, including the number of patients in each study, the number of raters, the median and/or mean NIHSS and DWI-ASPECTS of included patients, interrater vs. intrarater agreement measures (intraclass correlation coefficient and unweighted/weighted kappa statistics), information on dichotomization or trichotomization of the DWI-ASPECT score, and the MRI Tesla values. The degree of reliability using ICC (intraclass correlation coefficient) or kappa was determined as: poor agreement (0 to 0.20), fair agreement (0.21 to 0.40), moderate agreement (0.41 to 0.60), strong agreement (0.61 to 0.80) or excellent agreement (0.81 to 1).

## Results

A total of 3,658 titles and abstracts were screened, and 38 articles were initially included for full text review. From screening the references’ list of included eligible studies, only one additional study meeting our inclusion criteria was found, resulting in 39 articles included for full text review. Twenty articles were excluded after full text review as they did not meet inclusion criteria, resulting in 19 articles included for data collection and analysis. The PRISMA flow diagram is provided in Figure 1. A list of articles is provided in our Supplementary Table 2.

**Figure 1:**
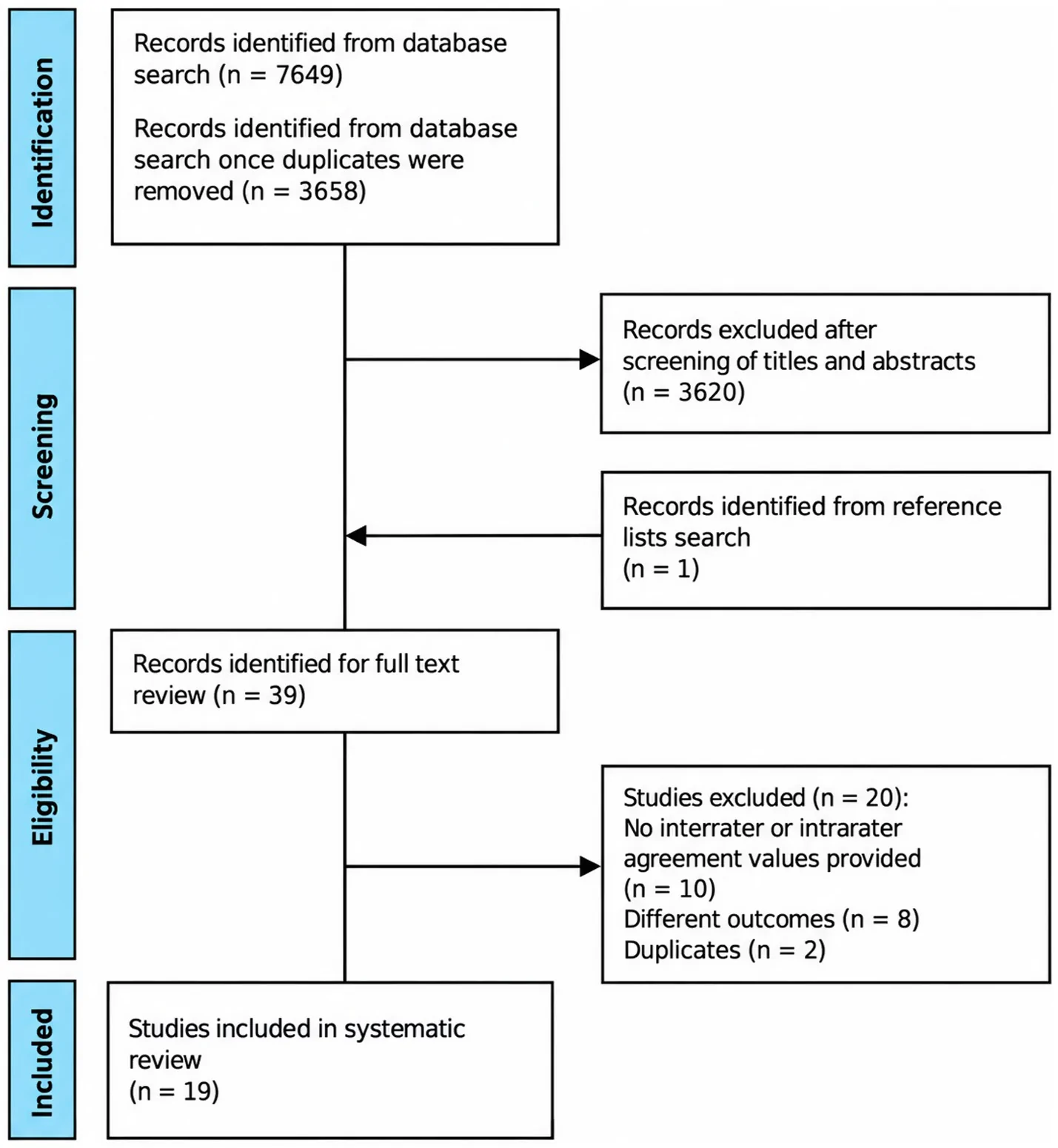
PRISMA flow diagram.

In our included articles, reporting was often incomplete: methods, populations, and results varied widely. (Tables 1, 2) The mean number of patients in the studies was 130.11 ± 84.23, ranging from 30 to 309 patients. The mean number of raters per study was 5.21 and ranged from 2 to 34, though only 3 studies had more than 5 raters. The number of raters for interrater agreement measures ranged from 2 to 34 and from 2 to 10 for intrarater agreement measures. Six (33.33%) interrater agreement studies had more than 2 raters and only 1 (50%) intrarater agreement study had more than 2 raters. Studies also used MRI scanners of varied Tesla (T) strengths across the studies: 1.5 T MRI images were used in 8 studies, 3 T MRI images in 4 studies, one study used both 1.5 T and 3 T MRI images, and 6 studies did not disclose information on the MRI magnetic field values.

**Table 1:** Interrater agreement values and study characteristics.

Study title	Number of patients	NIHSS median (IQR) or mean (±SD)	DWI-ASPECTS median (IQR) or mean (±SD)	MRI tesla value	Number of raters	Dichotomized or trichotomized groups	Interrater agreement value	95% CI
Interrater agreement - Global DWI-ASPECTS - ICC								
Yoshimoto 2019	198	21 (14–27)	5 (3–8)	3	2	—	0.766	0.693–0.786
Manceau 2018	82	18.4 ± 5.4	—	—	2	—	0.96	0.95–0.97
Albers 2019	65	19 (16–23)	3 (2–4)	—	2	—	0.72	—
Cheng 2021	309	—	5 (2–7)	1.5	2	—	0.954	0.943–0.963
Bing 2022	80	9.5 (6–16)	6.35 (SD ± 2.44) and 6.48 (SD ± 2.44)*	3	10	—	0.853	0.798–0.896
Kellner 2019	96	—	—	—	2	—	0.86	—
McTaggart 2015	74	17 (12–20)	—	1.5	2	—	0.867	—
Ha 2026	74	15 (10–18)	7 (5–9)	3	2	—	0.876	0.810–0.920
Interrater agreement - Global DWI-ASPECTS - Unweighted Kappa								
Rangaraju 2015	213	15 (11–18)	5 (2–7)	—	2	—	0.7	—
Fahed 2018 (1)	259	—	7 (5–8)	1.5	4	—	0.291	0.222–0.361
Kawano 2013	62	14 (5–31)	9 (5–10)	1.5	2	—	0.4851	—
Kawano 2012	164	14 (0–40)	8 (0–10)	3	3	—	0.434	—
Fahed 2018 (2)	30	17.5 (13.0–20.75)	7 (5–8)	1.5	18	—	0.17	0.14–0.21
Kellner 2019	96	—	—	—	2	—	0.33	—
Ducroux 2019	41	11 (5–17)	8 (6–9)	1.5	34	—	0.325	0.276–0.387
Mitomi 2014	130	12 (7–19)	7	—	2	—	0.76	—
Interrater agreement - Global DWI-ASPECTS - Weighted Kappa								
Fahed 2018 (1)	259	—	7 (5–8)	1.5	4	—	0.607	0.555–0.659
Yoshimoto 2019	198	21 (14–27)	5 (3–8)	3	2	—	0.985	0.981–0.989
Singer 2009	217	14 (9–17)	6 (5–8)	1.5	2	—	0.441	0.373–0.509
Butcher 2008 (experienced)**	35	—	—	3	2	—	—	—
Butcher 2008 (inexperienced)**		11 (4–25)	—	3	2	—	—	—
Lassalle 2016	232	14 (7–19)	7 (6–8)	1.5	2	—	0.96	0.91–1
Ha 2026	74	15 (10–18)	7 (5–9)	3	2	—	0.874	0.793–0.923
Interrater agreement - Dichotomized DWI-ASPECT - Unweighted Kappa								
Fahed 2018 (1)	259	17.5 (13.0–20.75)	7 (5–8)	1.5	4	0–7 vs. 8–10	0.666	0.571–0.761
Sakai 2020	111	4 (2–7)	9 (6–10)	—	2	0–6 vs. 7–10	0.663	—
Bing 2022	80	9.5 (6–16)	6.35 (SD ± 2.44) 6.48 (SD ± 2.44)*	3	10	0–6 vs. 7–10	0.75	—
Fahed 2018 (2)	30	17.5 (13.0–20.75)	7 (5–8)	1.5	18	0–5 vs. 6–10	0.62	0.48–0.75
Fahed 2018 (2)	30	17.5 (13.0–20.75)	7 (5–8)	1.5	18	0–6 vs. 7–10	0.68	0.55–0.79
McTaggart 2013	74	17 (12–20)	—	1.5	2	0–7 vs. 8–10	0.73	0.57–0.89
Interrater agreement - Trichotomized DWI-ASPECT - Unweighted Kappa								
Bing 2022	80	9.5 (6–16)	6.35 (SD ± 2.44) and 6.48 (SD ± 2.44)*	3	10	0–3 vs. 4–6 vs. 7–10	0.64	—
McTaggart 2013	74	17 (12–20)	—	1.5	2	0–4 vs. 5–7 vs. 8–10	0.68	0.53–0.83
Interrater agreement - Trichotomized DWI-ASPECT - Weighted Kappa								
McTaggart 2013	74	17 (12–20)	—	1.5	2	0–4 vs. 5–7 vs. 8–10	0.74	0.62–0.87

*The two DWI-ASPECTS mean ± SD values reflect two reading sessions separated in time with the same readers.

**Experienced readers are 2 stroke neurologists with >4 years of performing volumetric analysis and inexperienced readers were stroke fellows.

**Table 2:** Intrarater agreement values.

Studies	ICC or Kappa	95% CI
Bing 2022*	ICC 0.862	0.834–0.885
Kellner 2019	Rater A ICC 0.91	—
	Rater B ICC 0.93	—
	Rater A Kappa 0.47	—
	Rater B Kappa 0. 53	—

*The ICC is the mean ICC for 5 pairs of readers (including 1 senior neurologist and 1 senior radiologist). Individual ICCs for each reader were not available.

Overall, 37 measures of agreement were extracted from a total of 19 eligible studies. All 19 studies provided interrater agreement values (8 ICC values, 15 unweighted kappa values, 8 weighted kappa values) while only two studies provided intrarater agreement values. For interrater agreement, full scale ICC, unweighted kappa, and weighted kappa ranged from 0.72 to 0.96 (strong to excellent agreement), from 0.17 to 0.76 (poor to strong agreement) and from 0.441 to 0.985 (moderate to excellent agreement), respectively. Kappa values for dichotomized and trichotomized scores ranged from 0.62 to 0.75 (strong agreement) and 0.64 to 0.68 (strong agreement), respectively. (Table 1) For intrarater agreement, full scale ICC values ranged from 0.862 to 0.93 (excellent agreement), while kappa values ranged from 0.47 to 0.53 (moderate agreement). (Table 2).

Only five studies offered interrater data on dichotomized DWI-ASPECTS values, with three studies comparing agreement for DWI-ASPECTS ≤ 6 versus DWI-ASPECTS >6. One of these three studies also compared agreement for DWI-ASPECTS ≤5 versus DWI-ASPECTS > 5, and the two others compared agreement for DWI-ASPECTS ≤ 7 versus DWI-ASPECTS >7. Two studies offered interrater data on trichotomized DWI-ASPECTS values: one compared agreement for DWI-ASPECTS values 0–3 versus 4–6 versus 7–10, and the other compared agreement for DWI-ASPECTS values 0–4 versus 5–7 versus 8–10. No studies offered intrarater data on dichotomized or trichotomized DWI-ASPECTS values.

## Discussion

This systematic review aimed to evaluate clinicians’ interrater and intrarater agreement on DWI-ASPECTS. The literature revealed marked heterogeneity of populations, methods, results, and interpretations, precluding a meta-analysis. Specifically, our systematic review reveals heterogeneous interrater agreement measures that range from poor to excellent and a paucity of data on intrarater agreement measures. In the 2 studies that offered data on intrarater agreement, measures ranged from moderate to excellent.

The ASPECT score is an ordinal/categorical scoring system used to assess the degree of brain ischemia and to aid in making treatment decisions as it relates to mechanical thrombectomy. Traditionally, stroke guidelines have recommended treating patients with a large vessel occlusion and an initial ASPECTS ≥6 with thrombectomy (6–8). However, in light of recent positive randomized controlled trials demonstrating improved functional outcomes in the thrombectomy group compared to standard medical care for large ischemic strokes (DWI-ASPECTS 3–5) (13–16), there is an urgent need to evaluate and assess the reliability of DWI-ASPECTS for large strokes. Our systematic review reveals that not only are there limited data on interrater reliability for ASPECTS <6 [only 4 out of 18 studies], but of the limited studies that offered dichotomization or trichotomization comparisons of reliability for more severe ASPECTS subgroups (<6) (19–21), none looked explicitly at the reliability of DWI-ASPECTS 3–5 versus other categories. In addition, from the paucity of existing data on intrarater reliability of DWI-ASPECTS, none offered information on dichotomized or trichotomized values for more severe strokes. Therefore, there is a need for data on interrater and intrarater agreement in patients with DWI-ASPECTS <6, and in particular DWI-ASPECTS 3–5 (13–17).

The heterogeneity of results in our study mirrors the inherent limitations attached to the mathematical model of the DWI-ASPECTS scoring system. In essence, the numerical score provides an estimate of infarct core volume, a continuous measurement. Studies analyzing interrater and intrarater reliability of DWI-ASPECTS as a continuous variable using ICC resulted in mostly good to excellent agreement. However, infarct volume as a continuous variable can differ significantly for the same DWI-ASPECTS. This was demonstrated in the HERMES meta-analysis (22), which showed points were subtracted from different unequally weighted brain regions to obtain the same ASPECTS (23). At this time, it is not clearly defined how much volume loss is required in a specific region for it to register as a point on the ASPECTS. Given that disagreements in DWI-ASPECTS can lead to significant differences in prognostication (DWI-ASPECTS <6 versus DWI-ASPECTS ≥6) while other disagreements lead to little difference clinically, weighted kappa is a more appropriate measure of agreement in this case (24). Our study has demonstrated an interrater agreement for weighted kappa ranging from moderate to excellent agreement. On the other hand, unweighted kappa treats all disagreements equally and therefore would be a less clinically relevant measure for the DWI-ASPECTS (24). Not surprisingly, the highest level of heterogeneity in our study was observed with unweighted kappa values (ranging from poor to strong agreement). This heterogeneity in evaluating agreement for DWI-ASPECTS speaks to the difficulties encountered when using the score clinically and making decisions based on a categorical/ordinal scoring system that evaluates a continuous measure of volume loss.

Our study raises concerns similar to previous studies that assessed interrater and intrarater reliability of ASPECTS scoring on other imaging modalities. A previous systematic review and meta-analysis demonstrated low interrater agreement for CT-ASPECTS (9). While no review exists to our knowledge on the reliability of CTP-ASPECTS in acute stroke, individual studies have found higher degrees of interrater and intrarater agreement for perfusion deficits on CTP compared to CT-ASPECTS (25–27). In large strokes with ASPECTS <6, infarct volume ≥ 70 mL correlates to large cores and was found to be a better predictor of poor outcome than CT-ASPECTS ≤5 alone in patients undergoing thrombectomy within the first 6 h (28). These results speak to a need to better understand how infarct volume correlates to ASPECT scores in future studies.

Finally, DWI-ASPECTS is an ordinal scale meant to assess the presence or absence of ischemia at various locations (multiple categorical decisions) and transform it into a single numerical value. Thus, despite the perceived intrarater and/or interrater agreement over the single numerical value, raters may disagree on the exact locations of ischemia. It is also known that ASPECTS regions are unequal in their contribution to functional outcome. For example, in patients with a low DWI-ASPECTS (0–5) receiving thrombectomy, the involvement of M6 region in right-sided strokes and the internal capsule in left-sided strokes is associated with increased disability (29). This inherent heterogeneity in the ASPECTS regions adds a further layer of complexity when applying the scale clinically.

## Conclusion

In this systematic review of the literature on interrater and intrarater agreement for DWI-ASPECTS in acute ischemic stroke patients, we found marked heterogeneity in interrater agreement and a paucity of existing evidence for intrarater agreement. More specifically, data on interrater agreement for larger strokes (DWI-ASPECTS <6) are lacking, and essentially non-existent for intrarater agreement. Variations in agreement measures are directly related to the mathematical limitations of the score and need to be understood when applying the score clinically. Finally, more studies will be needed in the future for the evaluation of interrater and intrarater agreement of DWI-ASPECT in patients with a large core infarct.

## Data Availability

The original contributions presented in the study are included in the article/Supplementary material, further inquiries can be directed to the corresponding author.
