# Supplementary material for: The reliability conundrum of DWI-ASPECTS in stroke assessment: a systematic review of agreement on DWI-ASPECTS in anterior circulation stroke
Source: Front Neurol. 2026 Oct 23;17:1900599. doi: 10.3389/fneur.2026.1900599 (PMC13739564; doi:10.3389/fneur.2026.1900599)
Supplement: Supplementary file 1 [file Supplementary_file_1.docx]

**Supplementary Table 1.** Search strategy Jan 1^st^, 2000, to September 9^th^, 2026*

| **Search CINAHL Database (EBSCO Interface)** | | |
| --- | --- | --- |
| Step | Question | Results |
| S1 | TI DWI-ASPECTS OR AB DWI-ASPECTS OR TI Baseline ASPECTS OR AB Baseline ASPECTS OR TI ASPECTS score* OR AB ASPECTS score* OR TI (Alberta N2 Stroke N2 Program*) OR AB (Alberta N2 Stroke N2 Program*) OR TI Alberta Stroke Program Early CT score OR AB Alberta Stroke Program Early CT score OR TI ASPECTS-DWI OR AB ASPECTS-DWI | 1246 |
| S2 | TI Alberta Stroke Program Early CT score OR AB Alberta Stroke Program Early CT score OR TI Alberta N3 Program* OR AB Alberta N3 Program* OR TI (ASPECTS N2 CT N2 score) OR AB (ASPECTS N2 CT N2 score) OR TI (Serial N2 ASPECTS) OR AB (Serial N2 ASPECTS) OR TI (ASPECTS N2 score*) OR AB (ASPECTS N2 score*) OR TI (Stroke N3 Computed Tomography Score) OR AB (Stroke N3 Computed Tomography Score) | 890 |
| S3 Jan 1st, 2000, to December 31^st^, 2023. | S1 OR S2 | **1416** |
| S3 Jan 1st, 2024, to September 9^th^, 2026 | S1 OR S2 | **149** |
| **Search EBM Reviews (OvidSP Interface)**   - Cochrane Database of Sytematic Reviews; ACP Journal Club; Database of Abstracts of Reviews of Effects; Cochrane Clinical Answers; Cochrane Central Register of Controlled Trials; Cochrane Methodology Register; Health Technology Assessment; NHS Economic Evaluation Database | | |
| Step | Question | Results |
| S1 | DWI-ASPECTS.tw,kw. | 17 |
| S2 | Baseline ASPECTS.tw,kw. | 42 |
| S3 | ASPECTS score*.tw,kw. | 67 |
| S4 | (Alberta adj2 Stroke adj2 Program*).tw,kw. | 166 |
| S5 | Alberta Stroke Programme Early CT score.tw,kw. | 5 |
| S6 | ASPECTS-DWI.tw,kw. | 1 |
| S7 | Alberta Stroke Program Early CT score.tw,kw. | 105 |
| S8 | (Alberta adj3 Program*).tw,kw. | 201 |
| S9 | (ASPECTS adj2 CT adj2 score).tw,kw. | 59 |
| S10 | (Serial adj2 ASPECTS).tw,kw. | 3 |
| S11 | (ASPECTS adj2 score*).tw,kw. | 192 |
| S12 | (Stroke adj3 Computed Tomography Score).tw,kw. | 36 |
| S13 Jan 1^st^, 2000, to December 31^st^, 2023 | Or / 1-12 | **330** |
| S13 Jan 1st, 2024, to September 9^th^, 2026 | Or / 1-12 | **161** |
| **Search EMBASE Classic + EMBASE Database (OvidSP Interface)** | | |
| Step | Question | Results |
| S1 | DWI-ASPECTS.tw,kw. | 245 |
| S2 | Baseline ASPECTS.tw,kw. | 209 |
| S3 | ASPECTS score*.tw,kw. | 592 |
| S4 | (Alberta adj2 Stroke adj2 Program*).tw,kw. | 1641 |
| S5 | Alberta Stroke Programme Early CT score.tw,kw. | 107 |
| S6 | ASPECTS-DWI.tw,kw. | 37 |
| S7 | Alberta Stroke Program Early CT score.tw,kw. | 1032 |
| S8 | (Alberta adj3 Program*).tw,kw. | 2001 |
| S9 | (ASPECTS adj2 CT adj2 score).tw,kw. | 764 |
| S10 | (Serial adj2 ASPECTS).tw,kw. | 18 |
| S11 | (ASPECTS adj2 score*).tw,kw. | 1762 |
| S12 | (Stroke adj3 Computed Tomography Score).tw,kw. | 290 |
| S13 Jan 1^st^, 2000, to December 31^st^, 2023 | Or / 1-12 | **2931** |
| S13 Jan 1st, 2024, to September 9^th^, 2026 | Or / 1-12 | **944** |
| **Search MEDLINE (PubMed Interface)** | | |
| Step | Question | Results |
| Total Jan 1^st^, 2000, to December 31^st^, 2023 | (((((((Serial ASPECTS[TIAB] OR DWI-ASPECTS[TIAB] OR Baseline ASPECTS[TIAB] OR ASPECTS score*[TIAB] OR Alberta Stroke Program*[TIAB]) OR(((((ASPECTS CT score hascommentin) OR DWI-ASPECTS hascommentin) OR Serial ASPECTS hascommentin))) OR ((((ASPECTS CT score hascommenton) OR DWI-ASPECTS hascommenton) OR Serial ASPECTS hascommenton))))) OR Alberta Stroke Programme Early CT score[Title/Abstract])) OR ASPECTS-DWI[Title/Abstract] OR Alberta Stroke Program Early CT score[Title/Abstract] OR Alberta Program [Title/Abstract]) | **1142** |
| Total Jan 1st, 2024, to September 9^th^, 2026 | Or / 1-12 | **576** |

**Total number of studies after duplicates were removed:** 3658 studies.

*The review was initially conducted from Jan 1^st^, 2000, to December 31^st^, 2023. We eventually extended from January 1^st^, 2024, to September 9^th^, 2026, hence why the results are separated by dates above. We have used the same search terms for both date ranges.

**Supplementary Table 2. List of included articles**

1. Fahed R, Ben Maacha M, Ducroux C for the ASTER Trial Investigators*, et al.* Agreement between core laboratory and study investigators for imaging scores in a thrombectomy trial. *Journal of NeuroInterventional Surgery* 2018;10:e30.

# 2. Yoshimoto T, Inoue M, Yamagami H, et al. Use of Diffusion‐Weighted Imaging‐Alberta Stroke Program Early Computed Tomography Score (DWI‐ASPECTS) and Ischemic Core Volume to Determine the Malignant Profile in Acute Stroke. Journal of the American Heart Association 2018; volume 8, issue 22.

3. Sakai K, Komatsu T, Iguchi Y, Takao H,Ishibashi T, Murayama Y
   Reliability of Smartphone for Diffusion-Weighted Imaging–Alberta Stroke Program Early Computed Tomography Scores in Acute Ischemic Stroke Patients: Diagnostic Test Accuracy Study
   J Med Internet Res 2020;22(6):e15893

# 4. Rangaraju S, Streib C, Aghaebrahim A, et al. Relationship Between Lesion Topology and Clinical Outcome in Anterior Circulation Large Vessel Occlusions. Stroke 2015; volume 46, number 7.

# 5. Kawano H, Hirano T, Inatomi Y, et al. Hyperacute-Phase Computed Tomography–Diffusion-Weighted Imaging Discrepancy and Response to Thrombolysis. Journal of Stroke & Cerebrovascular Diseases 2013; volume 22, issue 4.

# 6. Albers G.W, Wald M.J, Mlynash M, et al. Automated Calculation of Alberta Stroke Program Early CT Score: Validation in Patients With Large Hemispheric Infarct. Stroke 2019; volume 50, number 11

7. Manceau, P.-.-F., Soize, S., Gawlitza, M., Fabre, G., Bakchine, S., Durot, C., Serre, I., Metaxas, G.-.-E. and Pierot, L. (2018), Is there a benefit of mechanical thrombectomy in patients with large stroke (DWI-ASPECTS ≤ 5)?. Eur J Neurol, 25: 105-110.

8. Kawano, H., Hirano, T., Nakajima, M. *et al.* Modified ASPECTS for DWI including deep white matter lesions predicts subsequent intracranial hemorrhage. *J Neurol* 259, 2045–2052 (2012).

9. Cheng, X., Su, X., Shi, J. *et al.* Comparison of automated and manual DWI-ASPECTS in acute ischemic stroke: total and region-specific assessment. *Eur Radiol* 31, 4130–4137 (2021).

# 10. Bing F., Berger I., Fabry A., et al. Intra- and inter-rater consistency of dual assessment by radiologist and neurologist for evaluating DWI-ASPECTS in ischemic stroke. Revue Neurologique 2022; volume 178, issue 3

# 11. McTaggart R.A., Jovin T.G., Lansberg M.G., et al. Alberta Stroke Program Early Computed Tomographic Scoring Performance in a Series of Patients Undergoing Computed Tomography and MRI: Reader Agreement, Modality Agreement, and Outcome Prediction. Stroke 2014; volume 46, number 2

# 12. Fahed R., Lecler A., Sabben C., et al. DWI-ASPECTS (Diffusion-Weighted Imaging–Alberta Stroke Program Early Computed Tomography Scores) and DWI-FLAIR (Diffusion-Weighted Imaging–Fluid Attenuated Inversion Recovery) Mismatch in Thrombectomy Candidates: An Intrarater and Interrater Agreement Study. Stroke 2017; volume 49, number 1

# 13. Singer O.C., Kurre W., Humpich M.C., et al. Risk Assessment of Symptomatic Intracerebral Hemorrhage After Thrombolysis Using DWI-ASPECTS. Stroke 2009; volume 40, number 8

# 14. Kellner, E., Reisert, M., Kiselev, V., Maurer, C., Urbach, H., & Egger, K. (2019). Comparison of automated and visual DWI ASPECTS in acute ischemic stroke. *Journal of Neuroradiology*, *46*(5), 288-293.

# 15. Butcher K., Parsons M., Allport L., et al. Rapid Assessment of Perfusion–Diffusion Mismatch. Stroke 2007; volume 39, number 1

# 16. Ducroux, C., Fahed, R., Khoury, N., Gevry, G., Kalsoum, E., Labeyrie, M., Ziegler, D., Sauve, C., Chagnon, M., Darsaut, T., & Raymond, J. (2019). Intravenous thrombolysis and thrombectomy decisions in acute ischemic stroke: An interrater and intrarater agreement study. *Revue Neurologique*, *175*(6), 380-389.

# 17. Lassalle L., Turc G., Tisserand M., et al. ASPECTS (Alberta Stroke Program Early CT Score) Assessment of the Perfusion–Diffusion Mismatch. Stroke 2016; volume 47, number 10

# 18. Mitomi M, Kimura K, Aoki J, Iguchi Y. Comparison of CT and DWI findings in ischemic stroke patients within 3 hours of onset. J Stroke Cerebrovasc Dis. 2014 Jan;23(1):37-42.

# 19. Ha, H. J., et al. “Validation of a Novel Automated Alberta Stroke Program Early Computed Tomography Score (ASPECTS) Software: Reliability, Magnetic Resonance Imaging (MRI) Correlation, and Early Ischaemic Core Discrimination.” *Clinical Radiology* [England], vol. 92, no. 107138, January 2026
